# Association of Coenzyme Q10 with Premature Ovarian Insufficiency

**DOI:** 10.1007/s43032-022-01136-1

**Published:** 2022-12-05

**Authors:** Linjuan Ma, Xiaojun Li, Chunming Li, Peiqiong Chen, Yibing Lan, Yizhou Huang, Wenxian Xu, Jianhong Zhou

**Affiliations:** 1 Department of Gynecology, Women’s Hospital, Zhejiang University School of Medicine, Hangzhou, 310006 China; 2 Lishui Central Hospital, Lishui, 323000 China

**Keywords:** Coenzyme Q10, Oxidative stress, Premature ovarian insufficiency

## Abstract

The aim of the study was to analyze the relationship between levels of coenzyme Q10 (CoQ10) and the risk of premature ovarian insufficiency (POI). In this cross-sectional case–control study, 32 women with POI and 58 women with normal menstrual cycles were recruited. The serum levels of follicle-stimulating hormone (FSH), luteinizing hormone (LH), anti-Müllerian hormone (AMH), CoQ10 and total cholesterol were measured. The association of CoQ10 with POI was assessed using binary logistic regression analysis. The CoQ10/total cholesterol ratio was significantly lower in the women with POI than in the women with normal menstrual cycles (120.94 ± 25.35 nmol/mmol vs 138.97 ± 39.19 nmol/mmol, *P* = 0.021). The serum CoQ10/total cholesterol ratio was inversely associated with POI (the unadjusted odds ratio (*OR*) = 0.984, 95% *CI*: 0.970–0.998, *P* = 0.027). The same trend was found after adjusting for confounding factors (such as age, body mass index, annual household income and education) (*OR* = 0.976, 95% *CI*: 0.956–0.996, *P* = 0.020). The serum CoQ10/total cholesterol ratio was inversely associated with POI, indicating that antioxidant deficiency may be a risk associated with the development of POI. CoQ10 may be a protective factor for ovarian tissue.

## Introduction

Premature ovarian insufficiency (POI), also known as primary ovarian insufficiency, is a clinical syndrome associated with the loss of ovarian function in women before 40 years of age, characterized by amenorrhea or menstrual irregularity for more than four months, hypoestrogenism and hypergonadotropism (two serum follicle-stimulating hormone (FSH) levels obtained at least 4 weeks apart > 25 IU/L) [1]. The incidence of POI is approximately 1% of the general population. Women with POI not only suffer from clinical complications associated with estrogen deficiency, such as osteoporosis, cardiovascular diseases, and cognitive impairment [2], but also endure the tremendous psychological pressure of infertility [3]. The causes of POI include chromosomal abnormalities [4], autoimmunity [5], iatrogenic factors [6], metabolic disorders [7], virus infections [8], environmental pollutants [9] and so on, but for more than 75% of the cases, the cause remains a mystery [10]. Recently, oxidative stress (OS) and high levels of reactive oxygen species (ROS) have been demonstrated to be correlated with POI [11].

CoQ10, as a lipid-soluble benzoquinone present in nearly all cell membranes, is an essential electron transporter in the mitochondrial respiratory chain necessary to produce cellular energy. CoQ10 is a potent antioxidant in cellular metabolism via its regenerating other antioxidants, scavenging free radicals and inhibiting the oxidation of lipids, proteins, and DNA [12]. Other effects of CoQ10 include motivating cell growth, inhibiting cell death, and maintaining the stability of the cell membrane [13]. CoQ10 synthesis deficiency is associated with certain clinical disorders in high-energy-consuming tissues, including endocrine glands, skeletal muscles, and the nervous system [14]. The level of CoQ10 has been reported to decrease in several tissues with aging [15] and in some pathological conditions with OS, such as diabetes, Alzheimer’s and prion diseases [16]. The presence of CoQ10 has been detected in human follicular fluid [17]. In addition, in mice, CoQ10 supplementation has been demonstrated to preserve ovarian reserve and improve mitochondrial performance in oocytes and ovulation rates [18, 19], but whether this association applies to human beings remains unclear. In this cross-sectional study, the potential relationship between the CoQ10 level and the odds of POI was assessed using logistic regression models.

## Materials and Methods

### Patients

Thirty-two women with POI and 58 women with normal menstrual cycles were recruited from the Department of Obstetrics and Gynecology at Women’s Hospital, School of Medicine, Zhejiang University (Hangzhou, Zhejiang, China) from December 2014 to May 2017. Diagnosis of POI was performed according to the European Society of Human Reproduction and Embryology (ESHRE) guidelines [20]: oligomenorrhoea or amenorrhoea for at least 4 months and an increased FSH level > 25 IU/L on two occasions > 4 weeks apart below the age of 40. The control group consisted of healthy women below the age of 40 with long-term regular menstrual cycles without any hormonal medication who had a physical examination in our hospital. Women with a history of chronic diseases (liver diseases, renal diseases, hypertension, cardiovascular or cerebrovascular diseases, and congestive heart failure), autoimmune diseases (thyroid diseases, adrenal diseases, and diabetes), hysterectomy, oophorectomy, chemotherapy, radiotherapy, hormone replacement therapy, oral contraceptives, pregnancy, breast-feeding, use of other drugs such as lipid-lowering agents, antihypertensive drugs, beta-blockers or tricyclic antidepressants, or supplementation with vitamins, CoQ10, and others were excluded from the study.

The Ethics Committee of Women’s Hospital of Zhejiang University approved this case–control study (Ethics approval number: 2015021). Informed consent was obtained from all participants.

### Measurements

The age, height, weight, occupation, education, and annual household income of each participant were recorded. The height and weight were measured by a professionally calibrated device. The body mass index (BMI) was calculated by the weight in kilograms divided by the height in meters squared.

Fasting venous blood samples were obtained from the antecubital vein of all participants. The control group’s blood samples were collected during the early follicular phase of the menstrual cycle (from days 1 to 5 of the menstrual cycle). All blood samples were centrifuged to separate the serum within 30 min after collection. All serum samples were frozen at − 80 °C until further analysis. The serum levels of FSH, LH, and AMH were measured using an automated Roche Modular Analytics E170 immunoassay system (Roche Diagnostics, Mannheim, Germany). Serum CoQ10 levels were measured by ultrahigh-performance liquid chromatography tandem mass spectrometry. To normalize the CoQ10 level, the total cholesterol levels were measured by using colorimetric analysis, and the CoQ10 levels, adjusting for total cholesterol, were calculated.

### Statistical Analysis

SPSS (Statistical Package for Social Science) 17.0 software was used for statistical analysis. Continuous variables were expressed as the mean ± standard deviation (SD) or median (interquartile range). The continuous variables with a normal distribution were compared by Student’s *t*-test. The Mann–Whitney *U* test was used if the continuous variables were non-normally distributed. Data for categorical variables were shown as the number and percentage of subjects. Categorical variables were compared by chi-square test. The correlations between each variable and the POI were assessed by the Spearman non-parametric correlation analysis. The association between the total cholesterol-adjusted CoQ10 levels and POI was further assessed by binary logistic regression analysis. The results were considered statistically significant if two-tailed *P*-values < 0.05.

## Results

The general characteristics of the participants are shown in Table 1. There were no significant differences in age at enrollment or body mass index between the case group and the control group (*P* = 0.615 and *P* = 0.581, respectively). Women in the control group had a significantly higher annual household income and education than those in the POI group (both *P* < 0.001).

**Table 1:** General characteristics of the patients and the controls

Characteristics	POI group (*n* = 32)	Control group (*n* = 58)	*P*-value
Age at enrollment (years)			
< 20	1 (3.1)	0 (0.0)	0.615^a^
20–24	5 (15.6)	6 (10.3)	
25–29	4 (12.5)	14 (24.1)	
30–34	8 (25.0)	20 (34.5)	
35–39	14 (43.8)	18 (31.0)	
Median (*IQR*)	33.5 (8.25)	32 (7.75)	
BMI (kg/m^2^)			
< 18.5	6 (18.8)	9 (15.5)	0.581^b^
18.5–24.0	22 (68.8)	43 (74.1)	
≥ 24.0	4 (12.5)	6 (10.3)	
Mean ± SD	21.26 ± 2.72	20.94 ± 2.64	
Annual household income (RMB yuan)			
< 30,000	6 (18.8)	0 (0.0)	< 0.001^c^
30,000–100,000	17 (53.1)	8 (13.8)	
> 100,000	9 (28.1)	50 (86.2)	
Education			
Elementary school	10 (31.3)	1 (1.7)	< 0.001^c^
High school	11 (34.4)	4 (6.9)	
College	11 (34.4)	53 (91.4)	

Data are expressed as *n* (%) or mean ± SD, *P* < 0.05 means statistically significant

*POI* premature ovarian insufficiency, *BMI* body mass index, *SD* standard deviation

^a^Mann-Whitney *U* test

^b^Student’s *t*-test for independent samples

^c^Chi-square test

Table 2 shows that the FSH and LH levels in the POI group were significantly higher than those in the control group (74.70 ± 33.34 IU/L vs 6.58 ± 1.90 IU/L, *P* < 0.001; 40.35 ± 17.12 IU/L vs 3.95 ± 1.62 IU/L, *P* < 0.001). In contrast, the case group had significantly lower AMH levels than the control group (0.01 (interquartile range *IQR* = 0.01) ng/mL vs 3.15 (*IQR* = 2.795) ng/mL, *P* < 0.001). The levels of CoQ10 were slightly increased in the POI patients, compared to those in the healthy women (527.56 ± 112.82 µg/L vs 503.93 ± 145.64 µg/L), although the difference was not statistically significant (*P* = 0.429). The total cholesterol levels in the POI group were significantly higher than those in the control group (4.96 (*IQR* = 1.19) mmol/L vs 4.325 (*IQR* = 1.14) mmol/L, *P* < 0.001). The ratio of CoQ10 to total cholesterol was significantly lower in the POI group than in the control group (120.94 ± 25.35 nmol/mmol vs 138.97 ± 39.19 nmol/mmol, *P* = 0.021).

**Table 2:** Biochemical parameters of the two groups

Variables	POI group	Control group	*P*-value
FSH (IU/L)	74.70 ± 33.34	6.58 ± 1.90	< 0.001^a^
LH (IU/L)	40.35 ± 17.12	3.95 ± 1.62	< 0.001^a^
AMH (ng/mL)	0.01 (0.01)	3.15 (2.795)	< 0.001^b^
CoQ10 (µg/L)	527.56 ± 112.82	503.93 ± 145.64	0.429^a^
CoQ10 (nmol/L)	611.06 ± 130.67	583.69 ± 168.69	0.429^a^
Total cholesterol (mmol/L)	4.96 (1.19)	4.325 (1.14)	< 0.001^b^
CoQ10/total cholesterol (nmol/mmol)	120.94 ± 25.35	138.97 ± 39.19	0.021^a^

FSH, LH, CoQ10, and CoQ10/total cholesterol are expressed as the mean ± standard deviation; AMH, total cholesterol are expressed as median (interquartile range); *P* < 0.05 means statistically significant

*POI* premature ovarian insufficiency, *FSH* follicle-stimulating hormone, *LH* luteinizing hormone, *AMH* anti-Müllerian hormone, *CoQ10* coenzyme Q10

^a^Student’s *t*-test for independent samples

^b^Mann-Whitney *U* test

Spearman non-parametric correlation analysis showed that POI was positively correlated with the levels of FSH (*r* = 0.829, *P* < 0.001), LH (*r* = 0.829, *P* < 0.001), and total cholesterol (*r* = 0.460, *P* < 0.001) but negatively correlated with education (*r* =  − 0.610, *P* < 0.001), annual household income (*r* =  − 0.603, *P* < 0.001), the levels of AMH (*r* =  − 0.782, *P* < 0.001) and the CoQ10/total cholesterol ratio (*r* =  − 0.255, *P* = 0.015). No correlations were found between POI and age (*r* = 0.053, *P* = 0.618), body mass index (*r* = 0.080, *P* = 0.451), or CoQ10 levels (*r* = 0.095, *P* = 0.375) (Table 3). As shown in Table 4, a binary logistic regression model was used to assess the relationship between the total cholesterol-adjusted CoQ10 and POI. The unadjusted odds ratio (*OR*) and 95% confidence interval (*CI*) were 0.984 (0.970–0.998), and the result was significant (*P* = 0.027). The same relationship was found in the adjusted model (adjusting for age, body mass index, annual household income and education) (*OR* = 0.976, 95% *CI*: 0.956–0.996, *P* = 0.020).

**Table 3:** The correlation between each variable and POI

Variables	Spearman correlation	*P*-value	*N*
Age	0.053	0.618	90
Education	− 0.610 **	< 0.001	90
Annual household income	− 0.603 **	< 0.001	90
BMI	0.080	0.451	90
FSH	0.829 **	< 0.001	90
LH	0.829 **	< 0.001	90
AMH	− 0.782 **	< 0.001	90
CoQ10	0.095	0.375	90
Total cholesterol	0.460 **	< 0.001	90
CoQ10/total cholesterol	− 0.255 *	0.015	90

*POI* premature ovarian insufficiency, *BMI* body mass index, *FSH* follicle-stimulating hormone, *LH* luteinizing hormone, *AMH* anti-Müllerian hormone, *CoQ10* coenzyme Q10

^**^Correlation is significant at the 0.01 level (2-tailed). *Correlation is significant at the 0.05 level (2-tailed)

**Table 4:** Association of CoQ10^a^ with POI in binary logistic regression models

Compound	Unadjusted model		Adjusted model^b^	
	*OR* (95% *CI)*	*P*-value	*OR* (95% *CI*)	*P*-value
CoQ10^a^ (nmol/mmol)	*N* = 90	0.027	*N* = 90	0.020
	0.984 (0.970–0.998)		0.976 (0.956–0.996)	

*CoQ10* coenzyme Q10, *BMI* body mass index

^a^Serum CoQ10 levels were adjusted for total cholesterol (nmol/mmol)

^b^The adjusted model included age, BMI, annual household income and education

## Discussion

The range for plasma CoQ10 concentration in humans is 0.5–2 µmol/L [21]. Palan et al. detected serum CoQ10 levels in 50 healthy premenopausal women (38.2 ± 7.9 years) and 33 healthy postmenopausal women (61.6 ± 10.6 years). The serum levels of CoQ10 in the postmenopausal women (0.74 ± 0.4 µmol/L) were significantly higher than those in the premenopausal women (0.59 ± 0.4 µmol/L); however, the difference was not statistically significant after normalization to cholesterol [22]. The serum levels of CoQ10 during the follicular phase in 10 healthy premenopausal women (28–44 years) were 0.50 ± 0.3 µg/dL in another study by Palan [23]. Chai et al. detected serum CoQ10 levels in 183 premenopausal women (34–47 years) (mean = 544 ng/mL, 95% *CI*: 511–578 ng/mL) [24]. Serum CoQ10 levels in the POI group were 527.56 ± 112.82 µg/L, and serum CoQ10 levels in the control group were 503.93 ± 145.64 µg/L in our study; therefore, our results are in accordance with the range of serum CoQ10 levels found in the above studies. CoQ10 and cholesterol share parts of a common synthetic pathway, and cholesterol is the main carrier of CoQ10 in serum [25]. Serum cholesterol is the strongest determinant of CoQ10 [21]. What is more, the synthesis of coenzyme Q was even decreased, following exposures of the cell cultures to cholesterol-poor serum. This indicates that the rate of coenzyme Q synthesis is dependent on the concentration of serum cholesterol. As shown in other published articles, they also adjusted the level of CoQ10 using CoQ10/total cholesterol ratio to ensure the accuracy of the research [26–28]. And CoQ10 may be used as a possible marker of in vivo oxidative stress [29]. Therefore, we should take into account the influence of cholesterol when we study CoQ10.

In this study, the adjusted serum CoQ10 levels were found to be significantly lower in POI patients. To the best of our knowledge, this is the first study to evaluate the relationship between CoQ10 and POI in humans. Previous studies on CoQ10 were mainly focused on reproduction and follicular development. Turi et al. demonstrated that CoQ10 exists in the human follicular fluid through using high-performance liquid chromatography on samples from 20 infertile women undergoing IVF-ET. The adjusted CoQ10 levels in the follicular fluids were higher in mature oocytes and high-grade embryos than in immature oocytes and low-grade embryos, respectively [17]. Palan et al. found that serum CoQ10 levels in the follicular phase were significantly lower than those in the luteal phase in healthy premenopausal women [23]. Therefore, CoQ10 may benefit follicular development. Gat et al. reported that the combination of dehydroepiandrosterone (DHEA) and CoQ10 significantly increased antral follicular count (AFC) and improved ovarian responsiveness in patients with decreased ovarian reserve receiving assisted reproductive technology (ART) [30]. Bentov et al. reported that women undergoing IVF who received CoQ10 supplementation showed reduced aneuploidy and increased pregnancy rates compared with women in a control group [31]. However, whether CoQ10 is an effective supplemental adjuvant therapy in ART still needs more prospective studies.

In an animal study, Ben-Meir et al. used conditional disruption of Pdss2 to interrupt CoQ10 synthesis in the oocytes of mice. Here, the mice exhibited a decreased number of ovulated oocytes and reduced ovarian reserve, compared to control mice shortly after the onset of puberty. In contrast, CoQ10 supplementation was shown to preserve ovarian reserve and improve mitochondrial function in oocytes and ovulation rates [19]. The deficiency of CoQ10 synthesis may induce ovarian insufficiency, which can be improved by CoQ10 supplementation. In a study by Ozcan et al., CoQ10 supplementation was reported to protect the ovarian reserve from OS-induced ovarian damage [18].

POI is the loss of ovarian function in women younger than 40 years old. Women with POI not only experience the distress of infertility but also undergo complications due to a long-term deficiency of estrogen, such as cardiovascular diseases, osteoporosis, sexual dysfunction and so on [32]. Therefore, exploration of the etiology, mechanism, and effective therapies of POI is of great importance. The etiology of POI is diverse, and most of the cases are idiopathic. The major mechanisms of ovarian dysfunction include follicle cell apoptosis, ovarian atrophy, cortical fibrosis, and OS and blood-vessel damage [33, 34]. To date, several studies have demonstrated that OS plays an important role in the development of POI [35, 36]. Levels of OS markers in patients with POI had been proven to be significantly higher than those in the control group in previous studies [11, 37]. OS occurs when the accumulation of ROS exceeds the antioxidant defense system. OS can induce mitochondrial DNA mutations and result in mitochondrial dysfunction [38]. Human oocytes contain the largest number of mitochondria. Mitochondrial DNA (mtDNA) copy number in the oocytes of women with ovarian insufficiency is lower than in those of women with normal ovarian cells [39]. Mitochondria, as the energy-transducing organelle of eukaryotic cells, produce adenosine triphosphate (ATP) through oxidative phosphorylation, providing energy for cell activity. Mitochondrial energy production is important in oogenesis and follicle maturation [40]. Mitochondrial dysfunction leads to a higher accumulation of ROS in ovarian tissues, which induces accelerated granulosa cell apoptosis and follicular atresia, finally diminishing ovarian reserve [41].

CoQ10, as a lipid-soluble antioxidant, is synthesized in the inner mitochondrial membrane and transported from complex I and II to complex III in the respiratory chain. CoQ10 can protect cells from free radicals and play an essential role in adenosine triphosphate (ATP) synthesis. CoQ10 deficiency can increase ROS production, result in OS and reduce respiratory chain activity [42]. CoQ10 deficiency can also reduce ATP synthesis and influence ovarian cell growth. CoQ10 is the only endogenously synthesized lipid-soluble antioxidant, and serum CoQ10 levels may correlate with human antioxidant defense. These results suggest that CoQ10 may be a new biomarker of POI.

In this study, we also found that women with a lower annual household income and education had a higher risk of developing POI. This higher risk may be due to differences in dietary structure and attitudes toward health. Additionally, women with POI had significantly higher total cholesterol levels, which means that they have an increased risk of developing atherosclerosis and coronary heart disease. There were still some limitations of our study. First, although the results of this study are encouraging, our relatively small sample size may have resulted in a shortage of statistical power. Second, CoQ10 exists in its reduced form (ubiquinol) and oxidized form (ubiquinone). Ubiquinol is the primary form of CoQ10 and is responsible for the antioxidant role of CoQ10 in vivo. In this study, we did not distinguish between the two forms of CoQ10. Third, the dietary intake of CoQ10 was not collected. Another limitation of our study was the uncertainty of whether the serum CoQ10 levels detected were a true reflection of the ovarian mitochondrial CoQ10 levels. In future studies, we may directly detect the levels of CoQ10 in ovarian tissues to evaluate the correlation between CoQ10 and ovarian function.

In conclusion, our study revealed a negative correlation between the CoQ10/total cholesterol ratio and risk of POI, which suggests that women with POI may have an increased oxidative burden and is of great value in its clinical application. Therefore, our findings suggest that supplementation with CoQ10 may protect or treat POI by eliminating oxidative stress. Further investigation involving a randomized control trial is needed to verify this hypothesis.

## Data Availability

The datasets used or analyzed during the current study are available from the corresponding author on reasonable request.
